# Misuse of Pregabalin: a qualitative study from a patient’s perspective

**DOI:** 10.1186/s12889-023-16051-6

**Published:** 2023-07-12

**Authors:** Louise Servais, Vincent Huberland, Lou Richelle

**Affiliations:** 1 Unité de Recherche en Soins Primaires ULB, Faculty of Medicine, Université Libre de Bruxelles, Route de Lennik 808, Brussels, CP 612 1070 Belgium; 2 Département de Médecine Générale, Faculty of Medicine, Université Libre de Bruxelles, Route de Lennik 808, Brussels, CP 612 1070 Belgium

**Keywords:** Drug misuse, Migration, High social risk, Qualitative study

## Abstract

**Introduction:**

The misuse of Pregabalin has been the subject of growing concern for several years. The effects sought are multiple and it is rarely taken as a single drug. It is most frequently used together with opioids, which may increase the risk of fatal overdose. In response to this emerging phenomenon, we seek to better understand the situation of misuse in Belgium and identify the people involved in it.

**Methodology:**

A qualitative study using semi-structured interviews with 20 participants who misuse Pregabalin was conducted throughout the French-speaking region of Belgium between August 2021 and January 2022. Recruitment mostly took place in addictions centers, shelters for migrants and homeless persons and primary care centers. We then conducted a thematic analysis with the help of Nvivo software.

**Results:**

A profile emerged, of young male users, immigrants, mainly first generation immigrants coming from North Africa. They had in common a challenging or even traumatic migration pathway and precarious living conditions in Belgian territory. Most of them had no stable income. They saw Pregabalin as enabling them to cope with their daily situation. All had psychiatric and/or somatic comorbidities for which they had apparently not recieved adequate medical care. This seemed to lead many of them to use Pregabalin as self-medication, for anxiety-depressive disorders and chronic pain, and it was sometimes initiated in their home country. Pregabalin was never used alone.

**Conclusion:**

This study has highlighted a rare and insufficient documented profile of Pregabalin misuse: self-medication among a group of first generation immigrants, most of whom have no previous history of opioid-related disorders. Measures should be taken to improve access to health and social care for this population, considering all their biopsychosocial vulnerabilities.

**Supplementary Information:**

The online version contains supplementary material available at 10.1186/s12889-023-16051-6.

## Introduction

Pregabalin (Lyrica ®) is a gabapentinoid, used as an add-on drug for partial epileptic seizures and also indicated in Belgium for neuropathic pain and generalized anxiety disorder in adults. The dosage is 300 to 600 mg per day [1]. It is increasingly prescribed *off-label* for chronic low back pain or radicular pain, despite a lack of evidence of efficacy for these indications [2].

On a European and international level, the misuse of this substance has been reported by several pharmacovigilance agencies over the last ten years [3–5], and tighter regulation of prescriptions is being introduced in several countries. It also appears to be a mortality risk factor in opioid overdose [5–7]. No official regulation is in place at the moment in Belgium, though Pharmacists and General Practitionners have been recently alerted of the misuse potential by official medical instances.

While experts agree in warning of this growing misuse, questions remain about the safety risks inherent to the molecule itself, whose lethality and addictiveness alone have not yet been demonstrated [8, 9]. The risk factors identified in US and European studies to date for Pregabalin misuse are: past or current opioid use, male gender, young age (between 18 and 45 years old), chronic diseases, history of addiction, medication for opioid use disorder, psychiatric comorbidities, multiple prescribers, low income [3, 5, 10].

The effects sought are euphoria, increased sociability, an energising or relaxing effect and a dissociative effect. Some patients use the drug to potentiate other substances, particularly opioids, or to cope with substance withdrawal [5, 7, 11]. “Self-medication" type use, for anxiolytic or analgesic purposes, has also been reported [12, 13].

In Belgium, this type of use has been observed among young men, immigrants, refugees, asylum seekers or irregular migrants, as illustrated in the latest report of the socio-epidemiologic organism Eurotox. Field associations have observed that the use of Pregabalin is probably related to a traumatic narrative and current living conditions that are difficult to sustain [14–16].

We wanted a better understanding of the phenomenon in the Belgian context, in order to improve the patient-centered care, since it is an emerging issue that seems to be challenging for some of the care providers confronted with it. We decided to take an interest in the patient's perspective. Our primary objective was to identify the background profile of the people who misuse Pregabalin in our context, and our secondary objective was to understand their motivations for using Pregabalin.

## Material and method

### Research terms and reflexivity

Qualitative interviews were conducted by the main author, who worked as a medical doctor (MD) in some of the centers where recruitment took place before and during the recruitment process. This process allowed a better understanding of the global situation and the appropriate skills to interact with this population. The researcher was known to be a MD by some of the participants, however she did not have a therapeutic relationship with any of them. It was clearly stated to participants that the interviews were for research purposes only, and that no Pregabalin prescription could ever be compiled by the researcher to the participants. The objectives and motivations of the study were clearly explained to the participants before asking for consent and conducting the interviews.

### Study design

We conducted a qualitative study using individual semi-structured interviews with participants, all of whom admit misuse of Pregabalin. “Misuse” is defined here as a supra-therapeutic dosage consumption, or a problematic use, whether according to clinical criteria or to the patient's own judgment. This method was selected to allow for diverse perspectives on this sensitive and complex subject.

The inclusion criteria for our study were, with no age limitation: Individuals who reported more than one episode of supra-therapeutic (> 600 mg/24 h) Pregabalin use in the three months prior to the study AND/OR Individuals who reported meeting the DSM-V or WHO ICD-11 criteria for substance use disorder regarding their Pregabalin consumption AND/OR Individual who sought help from a health care provider regarding their Pregabalin use in the year prior to the interview.

Individuals with a non-problematic (by their own judgement) usage of Pregabalin, within the therapeutic indications, and recommended dosages, were excluded from the study.

We contacted 50 organisations in Brussels and Wallonia, including addiction treatment centers, accommodation centers for persons with substance use disorder or homeless people, general practitioners, medical centers, and integrated primary health care centers. We aimed for a maximal variation sampling but were confronted with the difficulty of organising meetings with the patients in question, due to their high mobility, lack of structured schedule and reluctance to confide in a stranger on this delicate topic. Therefore, to ensure the recruitment, we decided to be present for one day per week at the site of one of the only low-threshold medical services prescribing Pregabalin in Brussels, allowing us to conduct 6 interviews in total. We also carried out 6 interviews via a Public Institution for Youth Protection (IPPJ) in a remote area of Belgium, which regularly receives unaccompanied foreign minors who misuse Pregabalin. The remaining respondents were recruited via their caregivers, with whom we made first contact: 2 came from a housing center for homeless people; 3 from various addiction treatment centers (2 in Brussels, 1 in Wallonia); 1 patient was met via a housing center for migrants; finally, 1 came via his general practitioner.

For the interviews, we drafted a guide that met the research objectives, based on the literature and experts’ consultation. We then pre-tested this guide with an individual meeting the study inclusion criteria before using it (See Additional file 1). The data concerning the subject's profiles were collected via closed and open questions, either before or after the interview, manually or orally (See Additional file 2).

Nineteen interviews were conducted, some in the presence of a socio-cultural mediator or an interpreter, from 08/02/2021 to 01/14/2022, until we reached external saturation of the qualitative data, meaning no new qualitative data was obtained when conducting new interviews.

The interviews were recorded using a phone and transcribed in extenso.

### Analysis and findings

The transcripts were analysed and coded by the principal author, using Nvivo software, following an iterative approach. We used a thematic analysis technique to identify patterns and similarities in the interviews [17]. It was decided to integrate the test interview (Interview n°0) into the corpus of interviews in order to enrich it, while taking into account its specificities during the data analysis. The quantitative data from the profile questionnaire was compiled using a Microsoft Excel spreadsheet and are shown in Table 1.

**Table 1:** Participant characteristics

Variables *N* = 20		
Place	Capital city	17
	Other city	3
Age (years old)	⩽18	5
	19–25	0
	26–35	9
	36–45	3
	46–55	1
	56–65	1
	Missing	1
Gender	Male	20
	Female	0
Birth country	Algeria	10
	Morocco	5
	Belgium	2
	Lybia	2
	Tunisia	1
Highest degree	< secondary school	12
	Secondary school	6
	Higher degree	0
Housing	Squat	6
	IPPJ	6
	Shelter	4
	Renting	2
	Owner	1
	Street	1
Income Source	Undeclared work	10
	Social welfare	4
	Theft	2
	None	2
	Deal	1
	Missing	2

### Ethics committee

Oral and written informed consent was obtained from each participant. The study was approved by the Erasme-ULB ethics committee on 07/14/2021, ref P2021/303 / CCB B4062021000156.

The study received the agreement of the Erasme-ULB hospital ethical board.

## Results

### Profile of respondents

The participants recruited were all men, aged 15 to 62, with a median of 30 years old. 9 of them were in the 26–35 age range, and 5 of them were minors. 17 participants resided in the capital city Brussels at the time of the interviews. Half of the respondents came from Algeria, 5 of them from Morocco. The majority did not finish high school. Only 3 of them had a private stable housing situation. Undeclared work was a source of income for half of the participants.

### Life course

#### Migration route

Eighteen of the 20 patients interviewed were first generation migrants from North Africa. They spoke about the negative aspects of this status, including the traumas and difficulties experienced during their multiple journeys and transitions to Belgium.

*"Yeah, I shocked, it's hard. […] You find the death (nervous laughter) […] Death, death. It's difficult, yeah. There are people who die, with us, he died. 5 days, no, no, no water, no, no food, and the sun. He has no appetite, 40 people, with the Africans, it's… Squadra Italia, he comes to us, the, the….the squadra from Italy he comes to take us from the sea." Interview 4*

Some mentioned regrets about their migration.

*"If he had known that it would be so complicated, he would have stayed in Algeria, because clearly he is… He was better off there, he felt much better and he lived near the sea, so clearly he didn't think it was going to be so complicated, he thought he was going to get a job more easily and so on, but Europe is changing and it's not at all what was explained to him, the fantasy he had.” Interview 12, hetero-anamnesis.*

#### Current living conditions

Most participants reported precarious living conditions in the host country. They were isolated, homeless, living in squats or on the streets. Undeclared work, often physically demanding, was cited as a means of livelihood. Conflict and violence were a daily occurrence. Several participants had spent time in prison, sometimes repeatedly.

*"Because I have a broken spine, and then I have to operate on an inguinal hernia because I used to do quite heavy work, warehousing, clarks, clarkist, getting pallets down here and so on. And then the hotel business, trays, cleaning big pots and pans and everything. Since I was 14, I've done nothing but work, I didn't stop and I had to run, for the family, my father died, I went to prison, I found myself in trouble, that's it. And it got out of hand.” Interview 2*

#### Comorbidities

##### Mental health disorders

All patients mentioned psychiatric comorbidities, such as depression or anxiety, or reported symptoms suggestive of them according to literature, such as anhedonia, clinophilia, sleep and appetite disorders and social isolation. Suicidal ideation or suicide attempts and self-harm were reported by some patients.

*"Sometimes I sleep, I sleep in the park, sometimes I sleep in the street. At that time I thought about… To commit suicide directly, definitively. That's why I take a lot of medicine, I have… How to say? […] Sometimes I eat a lot, the maximum! Really the maximum: Rivotril, Lyrica, Depakine to commit suicide definitively. Because I'm fed up, Doctor, I'm fed up. It's only been a year, only a year. […] Every time I think about suicide. Every time I go into hospital to commit suicide. I already have a file for suicide. 4 or 5 times suicided. […] Now I've cut. I cut all (lifts his shirt and shows me self-harm scars on his chest). I cut my belly. That's the problem." Interview 5*

Many patients have experienced natural or violent (sometimes related to war) deaths in their immediate family.

##### Somatic disorders

Patients reported significant physical traumas, such as road accidents or falls, sometimes while working for undeclared jobs. Several participants had undergone surgery. Pregabalin was initially prescribed to them for pain relief, either in Europe or in their home country, as a result of these traumas and operations.

*"And after the accident I take Lyrica for the painkiller because I had the operation here, in the disc (showing his back) […] Here, I broke here (showing his back), and in my head here. First I take Tramadol, after the doctor changed, he gives me Lyrica. Before I take Tramadol 200.”* Interview 4

They complained of chronic pain, for which Pregabalin was sometimes seen as indispensable.

##### Substance use disorders

All patients mentioned using at least one substance other than Pregabalin, mostly legal substances such as tobacco, alcohol and medication. Benzodiazepines, especially Clonazepam (Rivotril®), were the most commonly cited. Clonazepam was cited by 14 patients. 8 patients used alcohol. 5 patients used tobacco as the only substance other than Pregabalin. 3 patients mentioned Tramadol, of which only one was a current user. Concerning illicit substances, 10 patients used cannabis, 6 of them on a regular basis. 4 patients used cocaine. None reported having used drugs by injection and only 2 participants had a history of heroin use.

Some patients distinguished their use of Clonazepam from Pregabalin. They felt that Clonazepam would cause them more nervousness, interpersonal and legal problems and defined it as an "illegal drug", which was not always the case with Pregabalin.

*"With Rivotril, it gives problems with the police with everything. […] It's different. It's not like Lyrica. Lyrica you eat 5,6 it's not serious in the head. Rivotril 2,3 is good. Gives you problems."* Interview 19

### Substance

#### Substance meeting

Several participants had first used the substance in their country of origin, either Morocco or Algeria. The existence of widespread festive consumption in Algeria was mentioned by several participants.

*"Yes, so it's really consumed by a large part in a festive way, really to get high, for the well-being side. And then it's complicated to stop. So he says that it's more present in Algeria than in Morocco. In Morocco they don't know about this substance at all, in Algeria it's more the doctors who prescribe it…". Interview 12*

Some participants increased their use of Pregabalin during their migration, while others started their use in Europe, either via prescription or via the black market. A minority of them started using Pregabalin to substitute other drugs such as Tramadol or benzodiazepines, which they considered to be more problematic in relation to their substance use history.

*"Because, me, I, well, I know myself, I'm an addict, if I start taking opioid painkillers, well, I'm going to get hooked […] I don't know what else I can say, at least about Lyrica. I mean. I know I have, I have… I took it because I had to, I thought it was a painkiller, because he had offered me Tramadol, and as it's a morphine derivative I refused."* Interview 15

#### Way of administration and supply

The oral route was the way of absorption for all the participants interviewed, although other practices were observed, such as nasal intake or smoking. The amount usually consumed varied from 600 to 2400 mg, with a 4–6 300 mg pills mean per day. Some patients reported occasionnaly consuming far greater volumes (up to 25 pills for one participant).

Most of the patients obtained their supplies, at least partially, on the black market. Many supplemented their daily 600 mg prescription with black market pills. The price on the black market was around one euro per capsule, with variations between cities and discounts for large purchases.

Some patients mentioned the ease of obtaining the substance, either on the black market or by prescription.

*"So in fact, generally, all the people who are on the street, homeless, generally they all have, they all have this drug, so we'll say it's easy to get Lyrica".* Interview 14

*"Well, legally, I have a psychiatrist who has very little difficulty in prescribing it, who doesn't seem to be aware that there is a market or that it is something he should be monitoring"* Interview 0

#### Sought effects

**Fig. 1:**
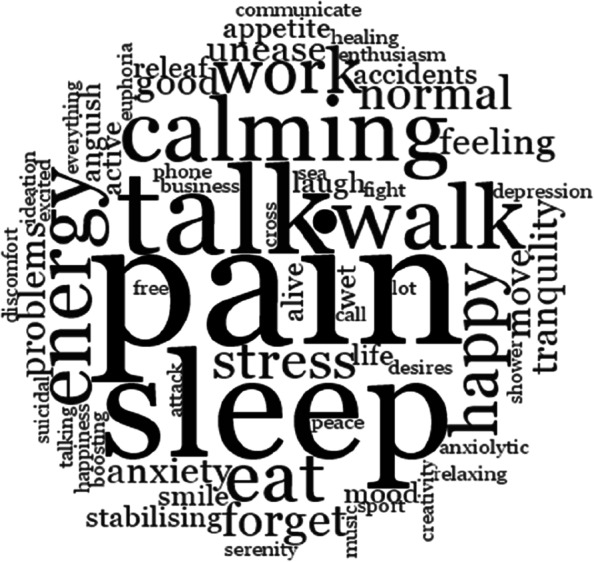
Word cloud, NVIVO "effects sought" codes frequency for all qualitative data collected

The effects sought were multiple, as illustrated in Fig. 1, showing the words the participants most frequently used when asked what effect they sought when consuming Pregabalin.

The overarching motivation for using Pregabalin appeared to be for enabling "normal" day-to-day functioning.

Mood effects were sought, including anxiolytic, antidepressant, euphoric, and sometimes suicidal ideation relief. An orexigenic effect was also cited by one participant.

Pregabalin appeared to be used for its entactogenic effect by the patients interviewed (to facilitate their social interactions).

*"If I had to eat Lyrica, I listen to music, I phone my mother, my father, I laugh all the time. Uh, I communicate with everything, peace, no fight."* Interview 6

An energising effect was sought by users, particularly for work or during their migratory journey.

*"Ok, so he really has an active life, when he has the Lyrica, so he knows how to go to work, he eats, he knows how to talk with his friends, he goes to the sport, end of story. He really has a more active life than when he doesn't have Lyrica."* Interview 12

*"In fact, in Turkey he started to use Lyrica because he was walking 30 kms a day to try to cross the border between Turkey and Greece. And at that time he consumes Lyrica to give him energy."* Interview 6

Paradoxically, Pregabalin was also used for sleeping by the same participants.

Analgesia was also a commonly sought effect.

Adverse effects were also reported, most commonly when taken in excess or when mixed with other substances. One notable adverse effect was amnesia which seemed to be linked to the dissociative effects of the substance. Feelings of drunkenness and palpitations were also reported.

*"Yes, it's already happened to me, ma'am, when I take, I forget quickly, it's like Alzheimer's. I swear, ma'am, it's not Alzheimer's. I swear, Madam, it's not Alzheimer's. But it's like Alzheimer's […] No, it's not, I mean, for example, sometimes I forget things. […] If I go over 10, if I go over 10 I get these symptoms, I forget things…".* Interview 7

#### Feeling of addiction to Pregabalin

Some patients mentioned their desire to control their consumption.

Some patients mentioned symptoms of “craving” while referring to the substance.

*" You're going to be searching like crazy, all day long, trying to find it. From morning to night.” Interview 2*

Most of the participants seemed to experience a tolerance effect, sometimes requiring a large increase in dose.

*"Ah, Lyrica is like that, madam. Because if for example I take, for example I take 3 today, so tomorrow if I take 3 it won't do anything. So you have to increase the dose. […] It's already happened to me some days, madam, where I take 26 pills and there's no effect, madam […] 26 pills and there's no effect, like that, there's no effect, so I have to drink alcohol like that afterwards.” Interview 7*

Participants reported a strong sense of psychological withdrawal, with symptoms such as unhappiness, fatigue, sleep and appetite disorders, clinophilia, anxiety, social isolation, nervosity, aggressivity, self-harm and suicidal ideation upon stopping Pregabalin.

*"So he takes risks for himself, he feels really, really bad, he doesn't recognise himself, he tells me, and he has a really hard time with other people. So even for him personally he doesn't eat, he doesn't drink, and he has a really hard time staying with others and staying in society, he has a really hard time with that. […] That's it, but then on a mental level. So he had a little reflection because he was ready to commit suicide, to do really stupid things because of the drug [During the withdrawal] Yeah, besides, there was some mutilation, he self-mutilated himself so much that it was bad.” Interview 12*

Physical withdrawal symptoms seemed to be of less importance, with night sweats and pain being the most commonly reported.

Few patients reported adverse consequences from their use of Pregabalin.

#### Perception of the substance

Few participants had clinical knowledge of the substance, neither its therapeutic indications, nor the risks associated with its use. Patients who had experienced excessive use without side effects considered it to be harmless.

*"Yeah never a problem. People said if I take 40 or 30 balls, I'll overdose. I never overdosed. […] It's not dangerous. No! Because I take huge drugs madam, he gave many, many times" Interview 16*

Pregabalin was considered by some respondents to be a medicine, a treatment, and not a drug. They made a distinction between themselves and people who use other substances.

*"… It's not me that… It's people who take drugs who do that, you see. I'm taking a treatment.”* Interview 10

*"No, cocaine is a drug and Lyrica Pregabalin is a medicine. Because when I don't take it, I stay like this, I don't talk, and I'm sad"* Interview 16

#### Weaning potential

Respondents seemed ambivalent about potential discontinuation of Pregabalin. Some of them had already made withdrawal attempts in the past. During these attempts, they reported having used other substances as substitutes such as alcohol, cannabis or benzodiazepines.

Participants cited the main obstacles to stopping consumption as their very precarious living conditions and the influence of peers.

*“When I've sorted out my problems I stop everything [laughs] -When you sorted out your problems? And what would have to be settled for you to be able to stop? What would you need to stop? -Same as everyone else. -Yeah? What does that mean? -Well, food, a wife, a house, and everything -A wife, a house, a family? -Everything, yeah. Holidays, buy a good car. That's it. -A good situation, right? -It's not…I don't want a Ferrari or something, a castle with the company and everything… Maybe… Less than you."* Interview n°10

Finding solutions for their mental health disorders and/or chronic pain was mentioned as a potential lever for considering stopping Pregabalin.

## Discussion

This study has explored an emerging category of misuse that has not yet been widely researched. To our knowledge, this is the first qualitative study on the profiles and experiences of people who misuse Pregabalin in Belgium. Data saturation was achieved around the 17th interview, where no new information was acquired, a small number of additional interviews were carried out to confirm this. The research was conducted according to best possible practices with the given constraints, and we believe that our main research question has been satisfactorily answered.

### Specific profile

The people we met had a profile similar to that previousely observed in some French studies [18–22], but different to what has been described in the international literature to date. Indeed, although they were using multiple substances, a minority of our participants were people with opioid use disorder, which is one of the risk factors most often described [5, 7, 10–13]. The majority of the patients interviewed were first-generation immigrants from North Africa, living in very precarious conditions on Belgian territory, which corresponds to the data in several limited reports on the specific situation in Belgium [14–16].

This consumption seemed to have been initiated and to have evolved at different times in the migratory pathway. For some, a progression from recreational use to substance use disorder seemed to take place during their migration. The imported aspect of this trend should not be overlooked, as the trafficking of pharmaceutical substances is a large illicit industry in many North African countries, and Pregabalin particularly is highly trafficked in the poor urban populations of Algeria [23]. For some, a switch from recreational use to substance use disorder seemed to take place during their migration. A certain trivialisation of use by our patients can also be explained by this phenomenon.

Concerning the sought effects, our participants mainly put forward effects that enabled them to cope with their current situation (arguing that Pregabalin would enable them to work, socialise, eat, sleep or combat ruminations or psychological malaise), as opposed to the search for a "high" or the potentiation of opioids or other substances, which are the sought effects that have been most often cited in other studies so far [5, 7, 11, 13, 24].

The easy access to Pregabalin on the black market, and its effects profile halfway between recreational and therapeutic and energizing, appears to have made it an “ideal” substance for these highly mobile patients whose survival depends on their ability to withstand the daily stressors to which they are subjected [25].

The lack of prescription regulation (especially for people with no health insurance) and the lack of awareness of prescribers of its misuse potential should also be mentioned as a facilitator of the current situation.

The fact that this phenomenon is found in a migrant population in our context can be explained by the vulnerability of migrants, refugees and asylum seekers to develop mental health disorders and addictions. The increased prevalence of risk factors in this population [26, 27] is linked to transit and travel conditions, loss of family and friendship networks, difficulties related to acculturation, poor living conditions in the host country and post-traumatic stress [28].

Legal status and stigma cumulation amongst these patients accelerates the deterioration of their health [29]. While contributing to their lack of help-seeking behaviour for their substance use and mental health disorders, it may also explain why it seemed important for them to differentiate themselves from people they see as “drug-users”, and their tendency to use licit or less stigmatized substances [30], such as medication, tobacco, cannabis and alcohol (though most of our participants had a Muslim background, reducing the risk of alcohol-use disorder [31]).

### Comorbidities

#### Mental health

Two main uses were identified, one related to mental health problems and the other related to chronic pain.

Regarding mental health, our hypothesis is that Pregabalin was used by the patients we interviewed as a form of self-medication for under-diagnosed anxio-depressive disorders for them. Indeed, they all reported typical symptoms of depression and anxiety. The more specific symptoms of adolescent depression, such as acting out and excessive substance use, were present in some of the minor patients encountered [32–34].

#### Chronic pain

In relation to chronic pain, Pregabalin also appeared to be used as a self-medication by patients to treat chronic pain, resulting from their multiple traumatic and surgical histories and precarious living conditions.

The increased difficulty of pain management with this vulnerable population cumulating biopsychosocial vulnerabilities and the willingness of caregivers to avoid using opioid analgesics or benzodiazepines, could explain why these patients were sometimes prescribed Pregabalin "off label" [4, 35].

### Limited access to health care

Their understanding of their health problems and pain seemed limited and may have prevented them from accessing adequate care. This is probably due to discontinuous care and lack of follow-up due to frequent migratory movements. A language and cultural barrier also probably deprived them of accessing to clear medical explanations [25]. The complexity of the multiple health systems with which they interacted during their journey and a lack of coordination between them potentially also contributes to the problem [27, 36].

Existing literature also reports limited access to health care for this population. This is despite their increased risk factors [27, 28, 36, 37], while they should benefit from better screening for anxio-depressive disorders and chronic pain, and have access to multidisciplinary, participatory and individualised care, adapted to their specific health needs [37–46].

### Limits

Despite our broad and multi-centered recruitment, our sample was mainly made up of men in very precarious living conditions. In practice, recruitment was only possible among low-threshold social and health services. As these services are less frequented by women and people who are more socio-economically integrated, there is a selection bias in our recruitment. This may also be seen as a strength: qualitative data about substance use disorders and mental health of homeless migrants is quite rare.

Our hypothesis, when designing this study, was that a second category of people would be encountered, consisting of people with poly-drug use, particularly opioid use. We only met two patients with this profile. An explanation could be the under-identification of Pregabalin use by the addiction centers, which generally do not prescribe the drug. Or Pregabalin misuse is maybe not very widespread among this group in Belgium at the time we conducted this study at the times. We also did not encounter patients coming from general practice, who seem to have a different profile than those in our study (patients with chronic pain, with more female patients in this population), according a recent cross-sectional study surveying Belgian GPs and social health services [47].

Regarding the interviews, several biases can be noted: Firstly, it is possible that some participants minimised their use or its negative impact problematic aspects (social desirability bias). Some people may also have been suspicious in their answers, as the interviews were conducted by a medical doctor seen as the prescriber of the molecule. A language barrier was also a hindrance to collecting good-quality data in some interviews. We were able to benefit from the help of a cultural mediator for some of the interviews, but this entailed a risk of distorting what was said or having the patient minimising the situation in front of a peer. Other interviews were conducted without the help of a cultural mediator, with rephrasing and gestures that may distort what the patient meant.

Finally, given the difficulty of meeting these patients in a structured way, many interviews were limited by external factors, such as the presence of other workers or spatial and temporal limitations.

Those elements prevented us from conducting the in-depth interviews and analyses expected.

### Perspectives

More studies, with a broader recruitment and sampling, are needed on the subject, in order to identify the potential other profiles of patients who misuse Pregabalin. It would also allow a better understanding of the problematic, in our context but also on an international scale.

## Conclusion

This study has identified a profile of people who misuse Pregabalin not previously described in the literature, except in small-scale and highly localised studies in France and Belgium. These people are young, male, first generation immigrants, mainly from North Africa, with very precarious living conditions in Belgium.

Their use appeared to be largely non-recreational and none of the patients sought to potentiate opioid use, in opposition to what has been most widely reported in the literature to date. Most of them seemed to misuse it as a coping strategy, in a context of anxio-depressive disorders, post-traumatic stress disorder and chronic non-neurogenic pain which are not in the therapeutic indications. Some, however, appeared to be self-medicating for a generalised anxiety disorder or for pain with a neuropathic component. In both cases, a diagnosis was rarely formally made and explained to the participant.

By focusing on the phenomenon of substance misuse, we were confronted with a much broader issue, which is the health of migrants in Belgium. Our work provides an insight into the lack of medical, psychological and social care experienced by this population, who nevertheless cumulate biopsychosocial vulnerabilities. To address this problem in a relevant way, it seems essential to focus on their specific health needs, responding adequately through combined, patient-centered and collaborative care approaches.

## Supplementary Information

**Additional file 1.** Interview grid.

**Additional file 2.** Profile questionnaire.

## Data Availability

All data generated or analysed during this study are included in this published article (and its supplementary information files). The dataset supporting the conclusions of this article is included within the article (and its additionnal files).
