# Supplementary material for: Misuse of Pregabalin: a qualitative study from a patient’s perspective
Source: BMC Public Health. 2023 Jul 12;23:1339. doi: 10.1186/s12889-023-16051-6 (PMC10337149; doi:10.1186/s12889-023-16051-6)
Supplement: Supplementary file 1 — Additional file 1. Interview grid. [file 12889_2023_16051_MOESM1_ESM.docx]

# Additional file 1: Interview grid

| **Theme: Main issue** | **Subtopics: Additional questions** | **Question of clarification** |
| --- | --- | --- |
| **Consumption history :**   - **Can you tell me about your consumption?** | - **What substance do you consume?** - **What about Lyrica?** |  |
| **Desired effect:**   - **Why do you use Lyrica?** | - **How does it feel?** - **Under what circumstances do you take them?** | - Can you give me some examples? |
| **Mode of consumption :**   - **How do you use Lyrica?** | - **Do you take it alone or with other products?** - **With what other products?** - **Do you take it instead of another substance?** - **How much do you usually consume?** - **Do you take it alone or with other people?** - **Where do you get it? Doctor, black market?** - **How much does it cost?** - **Are you aware of the use of Lyrica as a cutting substance?** |  |
| **Meet the substance:**   - **Under what circumstances did you first take it?** | - **When and where?** - **Why?** - **How did you get the drug for the first time?** | - Can you tell me a little more about it? |
| **Substance dependency :**   - **Do you feel you have an addiction to Lyrica?** | - **Do you sometimes feel a strong urge to take Lyrica?** - **Do you sometimes take more than you want or feel you can't stop yourself from taking it?** - **Do you feel physically ill when you haven't taken any for a long time?** - **Do you need to use more and more of it to get it to work for you?** - **Do you spend a lot of time during the day thinking about Lyrica and how to get it and use it?** - **Has taking Lyrica ever caused problems in your life?** | - Can you give me some examples? |
| **Substance knowledge :**   - **Do you think Lyrica is a dangerous drug ?** | - **Do you know why Lyrica is normally prescribed?** - **What are the side effects?** - **Do you know the risks of taking too much Lyrica?** |  |
| **Potential to stop :**   - **Do you plan to stop or reduce your use of Lyrica?** | - **What is the timeframe?** - **Under what conditions?** |  |

*Respondent's home institution :*

*Participant code :*
