# Supplementary material for: Misuse of Pregabalin: a qualitative study from a patient’s perspective
Source: BMC Public Health. 2023 Jul 12;23:1339. doi: 10.1186/s12889-023-16051-6 (PMC10337149; doi:10.1186/s12889-023-16051-6)
Supplement: Supplementary file 2 — Additional file 2. Profile questionnaire. [file 12889_2023_16051_MOESM2_ESM.docx]

# Additional file 2 : Profile questionnaire

**Profile questionnaire**

**Main place of care :**

**Gender :**

Male / Female / Other

**Age :**

**Nationality :**

**Languages spoken :**

Main :

Other :

**Main place of residence in the last 30 days :**

Home / Variable housing / Street / Institution / Prison

Other :

**Who did you live with most of the time during the last 30 days?**

Alone / In a couple / With my parents / With my children / With unrelated acquaintances

Other :

**Highest degree obtained :**

None / Primary / Secondary / Higher education / University /

Other :

**Source of income in the last 30 days :**

Salary / Unemployment / Scholarship / Sickness or invalidity allowance / Social assistance / Minimum income / Child allowance / Pension / No income

Other :

**Main work situation last 30 days :**

Regular employment / Casual employment / Unemployment / Studies/ Unable to work / Pension

Other :

**Migration history (open questions) :**

-Country of origin :

-Reason for leaving :

-Means of travel :

-Transit country :

-Milestones, Remarks :

*Respondent's home institution :*

*Participant code :*
